# A Novel Approach for Treating Benign Pleural Effusions—First in Human Application of a Diaphragmatic Pleuro-Peritoneal Valve

**DOI:** 10.1093/ejcts/ezaf448

**Published:** 2025-12-23

**Authors:** Ilker Iskender, Merjem Begic, Helmut Prosch, Clemens Aigner

**Affiliations:** Department of Thoracic Surgery, Medical University of Vienna, 1090 Vienna, Austria; Comprehensive Center for Chest Diseases, Medical University of Vienna, 1090 Vienna, Austria; Department of Thoracic Surgery, Medical University of Vienna, 1090 Vienna, Austria; Comprehensive Center for Chest Diseases, Medical University of Vienna, 1090 Vienna, Austria; Comprehensive Center for Chest Diseases, Medical University of Vienna, 1090 Vienna, Austria; Division of General and Paediatric Radiology, Department of Biomedical Imaging and Image-guided Therapy, 1090 Vienna, Austria; Department of Thoracic Surgery, Medical University of Vienna, 1090 Vienna, Austria; Comprehensive Center for Chest Diseases, Medical University of Vienna, 1090 Vienna, Austria

**Keywords:** chronic pleural effusion, diaphragm, pleuro-peritoneal valve, pleuro-peritoneal shunt

## INTRODUCTION

Herein, we present a novel treatment concept for persistent benign pleural effusion (BPE) using a pleuro-peritoneal valve inserted thoracoscopically into the central tendon of the diaphragm. Two patients were treated in an off-label individualised treatment attempt. This early report of a first-in-human application demonstrates the potential safety, feasibility, and efficacy of this device and treatment concept in patients with chronic BPE.

## BACKGROUND

In case of BPE refractory to conservative treatment and requiring repeated punctures, symptom-oriented interventions are well established.^1^ Talc-pleurodesis is frequently used to obliterate the pleural space, however, talc may cause a restriction and is limited to unilateral situations in which the lung is fully expandable. A well-established alternative is the insertion of indwelling-pleural-catheters (IPC). These catheters drain the pleural fluid externally and are feasible for bilateral use, including in patients with trapped lungs. Disadvantages are a reduced quality of life, risk for infection and metabolic or electrolyte disorders due to protein and electrolyte loss.^2^ Several other options, such as pleuro-peritoneal or pleuro-venous shunts, have been investigated using a device with manually operated pumps.^3^ These systems dependent on patient compliance and are prone to spontaneous closure, which ultimately led to market withdrawal. An internal pleuro-peritoneal drainage would be ideal due to the large peritoneal surface available for fluid resorption. However, because abdominal pressure is physiologically higher than pleural pressure, spontaneous drainage is limited.^4^ A one-way valve mechanism may overcome this challenge.

## METHODS AND PATIENTS

**Figure 1.**
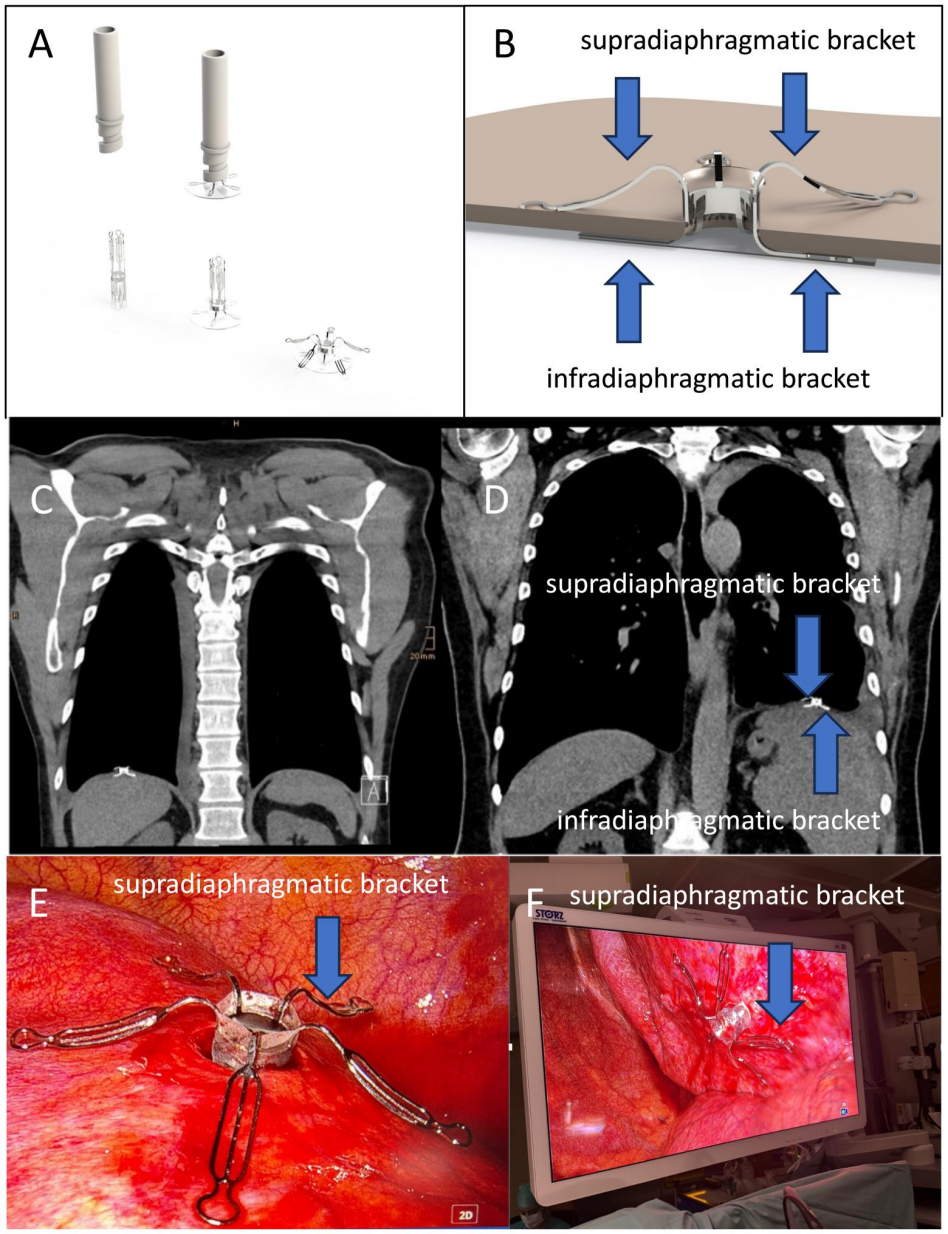
Deployment mechanism (A) and insertion situs of the diaphragmatic pleuro-peritoneal valve (B). The device has a diameter of 3 cm and consists of a thin infradiaphragmatic silicone sheet that integrates five one-way valves and a titanium scaffold with infra- and supradiaphragmatic brackets that retain the device in place and preclude obstruction of the pleural inflow by the lung. Frontal CT at follow-up (C and D) and intraoperative (E and F) views of the implanted valves in both patients. During implantation, the device is crimped into a custom-made delivery device, and the infra-and supradiaphragmatic portions are deployed individually.

Two patients with chronic symptomatic BPE were treated with a novel diaphragmatic pleuro-peritoneal valve (Medivio, Germany), as an off-label, individualized treatment-attempt under institutional oversight. No clinical approval for general use of the device exists. Written informed consent from both patients and local ethics-committee approval (2033/2025) was obtained. The first patient was a 33-year-old male with a history of mediastinal germ-cell-tumour resection including SVC-replacement with separate grafts to the brachiocephalic and innominate veins. Postoperatively, he initially developed a right-sided chylothorax followed by delayed thrombosis of the vascular grafts. Conservative management included anticoagulation, diuretics, a low-fat-diet, and repeated thoracentesis. Persistent serous bilateral BPE and exertional dyspnoea developed. Repeated bimonthly thoracenteses were required on the right side and at decreasing intervals on the left side. Malignancy and infection were ruled out. Talc-pleurodesis was deemed unsuitable due to bilaterality of effusions and the patient declined IPC placement due to limitations in quality of life and functional performance. Thus, right-sided diaphragmatic pleuro-peritoneal valve insertion was selected as an individual treatment attempt. Valve insertion was performed by a biportal VATS-approach using a custom-made punch to create a hole in the central tendon of the right diaphragm followed by valve placement with a dedicated delivery device (Figure 1). Surgery time was 71 min with no relevant blod-loss. A 20 F chest drain was removed on postoperative day 1 and the patient was discharged postoperative day 2.

The second patient was a 63-year-old male, who underwent a liver transplantation in 2013 for alcohol-related liver-cirrhosis. During routine follow-up a left-sided BPE with exertional dyspnoea and splenomegaly was diagnosed 10 years post-transplantation. Evaluation revealed a chronic transudative BPE without any signs of infectious or malignant causes. The patient benefited from repeated thoracentesis. Given his ongoing immunosuppressive therapy, the associated infection risk with IPC placement, and incomplete basal lung expansion, exploratory thoracoscopy with diaphragmatic valve insertion was recommended. The operative technique was comparable to the first case (Figure 1) with a surgery time of 42 min and a postoperative stay of 2 days. Follow-up was performed on days 10, 21, 60, 90 in both patients and on day 150 inpatient 1 only. Postoperative chest CT confirmed correct positioning of the valve on the diaphragm and minimal residual subvalvular pleural effusions < 1 cm slice thickness (Figure 1C and 1D). At 3 months follow-up, both patients reported no signs of dyspnoea and an improvement in quality of life by 15 points assessed using the EQ-5D-5 l. This improvement persisted in patient 1 at 5 months. Postoperative lung function in patient 1 showed a 10% increase in total lung capacity. No device or procedure related complications or adverse events occurred throughout the observation period. No further thoracocenteses were required and no signs of an inflammatory reaction were observed.

## DISCUSSION

Persistent BPE are a common problem and alters pleural pressure dynamics. A substantial proportion of patients require interventions to drain the BPE and to obliterate the pleural space. Both talc pleurodesis as well as IPC insertion are well-investigated treatment options.^2^

Alternatively, pleuro-peritoneal or pleuro-venous Denver shunts have been used, however were stopped due to associated complications, such as obstruction, pump-malfunctioning, infection, thrombosis and embolism.^3^

In both patients, talc-pleurodesis was not considered ideal, either because of initially bilateral effusions or insufficient lung expansion. Insertion of an IPC was declined due to concerns about quality of life and the risk of infection. Thus the decision for diaphragmatic pleuro-peritoneal valve insertion was made. As previously demonstrated by the use of other pleuro-peritoneal shunts the peritoneum has a larger absorption capacity compared to the pleura. The normal absorption rate is 1 ml/min capacity, however can reach up to 5 ml/min. Even though abdominal pressure normally exceeds intrathoracic pressure, dynamics in the respiratory cycle and postural changes lead to changes in this relation.^5^ Along with the gravitational forces these dynamics may enable fluid above the implanted valve to drain into the peritoneal cavity. Based on the presented observations it can be hypothesized that this concept is not dependent on patient compliance, requires only a single intervention, and appears to permit continuous passive pleuro-peritoneal drainage. Quality of life results are encouraging, however based on post-procedural EQ-5D-5 l assessment only must be interpreted with caution.

In summary, this “first-in-human” application of a novel treatment-concept using a non-CE marked diaphragmatic pleuro-peritoneal-valve in two patients with BPE as individualized treatment attempt demonstrates the potential safety, feasibility, and early efficacy of unilateral application. Results cannot be generalized and enrollment of additional patients, bilateral application and long-term follow-up are needed to demonstrate the potential benefits of this novel device in reducing healthcare-burden of patients with chronic BPE.

## Data Availability

All data will be shared upon reasonable request to the corresponding author.
